# Interest and learning in informal science learning sites: Differences in experiences with different types of educators

**DOI:** 10.1371/journal.pone.0236279

**Published:** 2020-07-23

**Authors:** Kelly Lynn Mulvey, Luke McGuire, Adam J. Hoffman, Eric Goff, Adam Rutland, Mark Winterbottom, Frances Balkwill, Matthew J. Irvin, Grace E. Fields, Karen Burns, Marc Drews, Fidelia Law, Angelina Joy, Adam Hartstone-Rose

**Affiliations:** 1 North Carolina State University, Raleigh, North Carolina, United States of America; 2 University of Exeter, Exeter, United Kingdom; 3 University of Cambridge, Cambridge, United Kingdom; 4 Centre of the Cell, Queen Mary University of London, London, United Kingdom; 5 University of South Carolina, Columbia, South Carolina, United States of America; 6 Riverbanks Zoo and Gardens, Columbia, South Carolina, United States of America; 7 Virginia Aquarium & Marine Science Center, Virginia Beach, Virginia, United States of America; 8 EdVenture, Columbia, South Carolina, United States of America; University of Hong Kong, CHINA

## Abstract

This study explored topic interest, perceived learning and actual recall of exhibit content in 979 children and adolescents and 1,184 adults who visited informal science learning sites and interacted with an adult or youth educator or just the exhibit itself as part of family visits to the sites. Children in early childhood reported greater topic interest and perceived learning, but actually recalled less content, than participants in middle childhood or adolescence. Youth visitors reported greater interest after interacting with a youth educator than just the exhibit, and perceived that they learn more if they interact with an educator (youth or adult). Participants in middle childhood recall more when they encounter a youth educator. Adult visitors reported greater interest after interaction with a youth educator than with the exhibit alone or an adult educator. They also perceived that they learn more if they interact with an educator (youth or adult) than just the exhibit and perceived that they learned more if they interacted with a youth educator than an adult educator. Results highlight the benefits of educators in informal science learning sites and document the importance of attention to developmental needs.

## Introduction

Much of the prior research on science interest and learning has centered on experiences in formal educational settings—classrooms and schools [1, 2]. However, youth spend the majority of their time outside of formal school environments [3]. Often youth have the opportunity during out of school time to engage in activities that might foster science interest and learning, with prior research demonstrating that these out-of-school-time science experiences lead to science interest and engagement [4, 5]. Likewise, adults frequently engage in science learning after their formal schooling ends, for instance through hobbies [6]. Museums, zoos, aquariums and other informal science learning sites (ISLS) function as rich sources of science content and as engaging spaces where learning might occur outside of the formal classroom environment [7].

The aim of the current study is twofold: 1) to explore developmental differences in the experiences of youth and adults in ISLS, in terms of perceived and actual learning as well as science interest; and 2) to examine what makes for an optimal learning experience in ISLS, with attention to comparing visits where one interacts with an educator (youth or adult) or just the exhibit material.

### Visiting and learning in informal science learning sites

There are several reasons for investigating learning and interest opportunities in ISLS. One reason is that ISLS draw large numbers of visitors annually. In particular, data from 181 science centers and museums globally in 2016 documented over 67 million visits to ISLS in one year, with only 15.2% of these visits were formal school groups or school trips, indicating that the vast majority of visitors to ISLS each year are selecting to visit during their leisure time [8].

Recent research has documented the benefits of out-of-school science experiences in shaping attitudes towards science [9, 10]. For instance, experiences in numerous high-quality informal learning environments are associated with growth in science learning outcomes, such as scientific reasoning [11]. Research on experiences in ISLS, in particular, has not yet fully explored the benefits of these experiences; much of the prior research has simply examined the overall benefit of many different types informal science experiences, for instance participating in research labs, looking at science websites, collecting items in nature, or doing science experiments at home [4, 5], but has not attended to particular benefits of each type of informal learning activity. However, some prior research has indicated that educators and the particular features of exhibits (for instance those that foster social interaction) in ISLS can play a particularly important role in visitor learning and engagement. For example, museum educators can perceive their role to be to “make a difference” for visitors [12]. Research demonstrates that interacting with an educator can provide scaffolding of the learning experience for visitors [13] and that the personal connection with an educator in a museum is centrally important [14]. Further, findings suggest that exhibits can foster visitor engagement, particularly if they allow for social interaction [15]. Research with families in ISLS has also clarified that adults can play an important role in shaping the experience at an informal learning site. Young children (4–6 years) engage in more discussion and testing of hypotheses if parents are asked to encourage their children to explain while visiting an exhibit and that they spend more time exploring if parents are encouraged to explore more with them [16]. Further, evidence suggests that when parents make more attempts to draw connections between their children’s prior knowledge and experiences and the exhibits they are exploring, children (ages 3–11 years) are more interested and attentive and when parents use more sense-making (discussing evidence, and scientific explanations, for instance), children are more conceptually engaged [17]. These findings indicate that simply visiting an ISLS alone will not necessarily foster interest and learning, but rather that the nature of the experience in that ISLS plays an important role in fostering interest and learning.

Much of the prior work documenting variations in outcomes from ISLS visits attends to family visits with a focus on the role that parents play [16–18]. Another large body of research explores outcomes of school visits with attention to the nature of the education that occurs [15, 16, 19, 20–22]. The current investigation explores family visits, but is distinct from the prior work on family visits which explores the role of parents as educators. Instead, the current project attends to the family unit: rather than exploring adults in terms of their role as parent-educators, we focus on their role as learners alongside their children with attention to their interactions to the exhibits and with youth and adult educators at these sites.

### Theoretical framework

The current study draws on Vygotsky’s Social Learning Theory [23, 24], which argues that we construct our learning in social environments. This theory also places importance on social opportunities to learn from those who are more knowledgeable than ourselves. Vygotsky posits that learning occurs when individuals are within their zone of proximal development, which is the space where one can move beyond what they are capable of with the support and scaffolding of a more knowledgeable other [23]. Theorists have argued that peers, those not much older than one’s self, may be particularly effective as more knowledgeable others [25]. Within the current study, we draw on this perspective in theorizing that the presence of an educator will foster those personal connections and social interactions that will be likely to create an optimal environment for learning and for promoting interest in the science topics explored at the ISLS.

However, we also expect that youth educators may be a particularly good learning facilitator for child and adolescent visitors in these ISLS, as youth educators (educators who are 14–18 years of age, often participating in teen docent programs at these sites) may be able to make more personally relevant connections and to build rapport with youth visitors than an adult educator (over 18 years of age). For child and adolescent visitors, we expect that there may be a particular benefit to working with a youth educator based on prior research on peer tutoring, which shows that youth peer tutors are effective in teaching other youth [26, 27]. Most of the prior research on youth learning from each other, however, has been conducted on peer tutoring or peer education in formal school settings or structured afterschool programs [28]. Research is needed to examine the benefits of learning from youth educators in ISLS. Some research has compared the impact of youth versus adult docents in providing guided school tours in library settings, demonstrating greater learning and satisfaction with tours guided by youth docents. Findings suggested that youth docents or educators provided tours which elicited more personally relevant connections to the content, and asked for more feedback from the youth with whom they interacted [29]. Therefore, building from these theoretical perspectives, we expect that visitors who interact with an educator (youth or adult) will learn more than those who do not (i.e., those who explore the exhibit unguided). Further, we also anticipate that for youth visitors there may be a particular benefit to interacting with a youth educator over an adult educator or no educator, because youth educators may be more likely to connect in relevant ways with youth visitors.

### Current study

The current study involved surveying adult, children and adolescent visitors to ISLS following their exploration of focal exhibits at these ISLS. All exhibits included static media that focused on a particular science topic and were, at times, staffed by education team members (youth or adult, depending on the day) who were trained to educate visitors on the same topic. Thus, we compared three conditions (no educator, youth educator, adult educator). Adults and youth aged 5–18 were invited to participate and completed a survey assessing their interest in the science topic of the exhibit which they visited, and their perception of how much they remembered from the exhibit. Finally, participants completed three age-appropriate multiple choice recall questions in order to assess how much they remembered from the exhibit. As we were unable to control for prior content knowledge and as perceived and actual learning may not always align, we included both perceived learning and a more explicit measure of recall. As data was collected in busy ISLS, the measures were kept very brief (single-item for perceived learning and topic interest, and three items for content knowledge). Our first hypothesis related to educator condition (Hyp 1): we expected that participants would learn more, perceive that they learn more and be more interested in the topic if they interacted with an educator (youth or adult) than if they just encountered the static media.

An additional focus of the current study involved developmental differences for the youth visitors. We expected that there might be differences in how youth in early childhood, middle childhood and adolescents responded to our measures. First, prior research demonstrates that children in early childhood often over-estimate their self-efficacy and perceived competence [30], thus we expected that children in early childhood might perceive that they learned more than youth in middle childhood or adolescence (Hyp 2). Next, as research has documented a decline in science interest across middle childhood and adolescence (see Osborne, Simon, & Collins [31] for a review), we expected that youth in middle childhood and adolescence would express lower interest in the science topics presented than would participants in early childhood (Hyp 3). In terms of learning content, we expected to observe an interaction between age group and education condition. Specifically, we expected that, with age, participants would get more items correct as they would have a greater wealth of prior knowledge on which to rely (Hyp 4). However, we also expected that older youth (middle childhood and adolescents) might learn more from youth educators than would participants in early childhood, as youth educators may be able to make more relevant prior connections to visitors closer in age to themselves (Hyp 5). In terms of the adult visitors, it was an open question whether they would benefit more from working with adult or youth educators.

## Methods

### Participants

Participants included 979 children and adolescents (59.8% female, 60.9% European-American or White British) and 1184 adults (72.6% female, 71.2% European-American or White British) who visited one of five ISLS located in the United States (US) and the United Kingdom (UK). The US sites consisted of a large zoological park and botanical garden (~1.5 million visitors annually), a medium-sized city-centered children’s museum (~200,000 visitors annually), and a large aquarium and marine center (~600,000 visitors annually), all located on the east coast of the US. The UK sites consisted of a large technology and engineering centered science museum (~240,000 visitors annually) located in a metropolitan area in the West Midlands and a small interactive biomedical science centre (~20,000 visitors annually) located in a research institute in a metropolitan area of the South-East of the UK. Youth participants were grouped into three age groups for analytic purposes: early childhood (*N* = 409, *M* = 6.77, *SD* = 1.03), middle childhood (*N* = 378, *M* = 9.94, *SD* = 0.823), and adolescence (*N* = 215, *M* = 13.67, *SD* = 1.63).

Participants were invited to participate in a brief survey after visiting a specified exhibit (see details in procedures) and each family was given a small electronic gift card or gift bag (worth £/$5) for their participation in the study. In the US this research was approved as exempt by IRB, however, participants and their parents were also given an informational letter notifying them about the research objectives. In the UK, ethical approval was received from IRB and informed consent was obtained.

### Procedure

The research team pre-selected exhibit sites at each ISLS where both adult and youth educators were often present to interact with and teach the visitors about the exhibit topic, but where the static exhibit media also communicated the same key information about the exhibit topic. For the purposes of this research, on data collection days the exhibit was staffed either by no educator, by an adult educator or by a youth educator and data collection was scheduled to ensure that we obtained data from visitors in each of these three conditions. Thus, visitors were not assigned to a particular condition, but all visitors visiting on a particular day were in the same condition (youth educator = 29.4%, adult educator = 21.2%, no educator = 49.4%).

Data were collected at more than one exhibit at the ISLS (21 exhibits in total). In the adult educator condition, the exhibit was staffed by a trained adult educator (aged 19+). In the youth educator conditions, the exhibit was staffed by a trained youth educator (14–18 years old). In the third condition, visitors experienced the exhibit without an exhibit educator and interacted only with the signage and interactive elements of the exhibit. Exhibits were selected so that equivalent information could be obtained from the signage and interactive elements as was shared by the youth and adult educators.

### Measures

All measures were developed for this study and wording was adjusted for American or British English, depending on the site location. Measures were kept brief given constraints on time due to the ISLS settings and different response scales were used for each item to assure attentional focus.

#### Topic interest

Participants were asked “How interested are you in the topic you just learned about?” (Likert scale, 1 = *not at all*, 5 = *a lot*).

#### Perceived learning

Participants were asked “How much did you learn from the exhibit?” (Likert scale, 1 = *nothing*, 6 = *a lot*).

#### Content recall

In order to assess recall of content, participants were asked content-based questions that were specific to the exhibit they visited. The wording of the questions was tailored to the age-group being surveyed. For example, in the question from a Komodo Dragon exhibit at the aquarium, children were asked, “Their small home range causes danger to the Komodo Dragons because…” and adolescents were asked, “Which of the following aspects of their small home range makes Komodo Dragons particularly vulnerable?” Each question had four multiple choice options with one correct response. Correct responses were given a score of 1 (incorrect responses were scored as 0) and scores were summed for possible total score of 0–3 for each participant.

### Data analytic plan

First, unconditional models including only exhibit were fit for topic interest, perceived learning and content questions correct, in order to assess the variance within and between exhibits. For youth, the inter-class correlations (ICC) for topic interest (.05) and perceived learning (.07) were small, and the ICC for content questions was large (.36). For adults, the inter-class correlations (ICC) were somewhat larger for topic interest (.14), and perceived learning (.09) and somewhat lower for content questions (.25). However, the design effects, which capture how much sampling error might be inflated due to the nested nature of the data [32] for all three variables of interest for both youth and adults were greater than 2.0, suggesting the importance of accounting for the nested nature of the data [32, 33]. Thus, accounting for a random effect of exhibit, models were estimated using the mixed command in SPSS Version 25 [34] following best practices for multilevel modeling in SPSS [35]. Analyses were conducted separately for the adult sample and for the youth. It is important to note that we were unable to account for the effect of parents on children’s learning and interest in the ISLS. For the youth sample, models including educator condition (youth educator, adult educator, no educator) and age group (early childhood, middle childhood, adolescence) as well as the interaction terms as fixed effects were tested with exhibit as a random effect. For the adult sample, models including educator condition (youth educator, adult educator, no educator) as a fixed effect and exhibit as a random effect were tested. We treated exhibit as a random effect because we selected a number of exhibits for this study from the larger population of possible exhibits (but did not sample from all exhibits). We selected to model educator as a fixed variable as we were interested in the average effect of educator across all exhibits. This is for a few reasons: 1) we believe, conceptually, that the different educator conditions should be equivalent across exhibits (for instance, there is no reason to expect that adult educators would be more effective at one particular exhibit than another) and 2) the conditions were designed so that the education experience was similar across exhibits (there were signs present providing content that was matched to what the educators were trained to present). Finally, educators at the sites are trained to provide interpretation at a number of exhibits and the training is similar across exhibits.

The equations are as follows.

### Children

In these equations, the outcome for the i^th^ visitor in the j^th^ exhibit is modeled as main effect of educator condition (γ_10_), the main effect of age group (γ_20_), and the interaction between educator condition and age (γ_30_) with γ_00_ as the overall mean and u_0j_ as the exhibit residuals and *e*_*0ij*_ as the individual residuals. This general equation was tested for each of the three dependent variables (Topic Interest, Perceived Learning and Total Content Questions Correct).

$$\gamma_{ij}=\gamma_{00}+\gamma_{10}EducatorCondition_{ij}+\gamma_{20}{\text{Agegroup}}_{\text{ij}}+\gamma_{30}EducatorCondition*{\text{Agegroup}}_{\text{ij}}+{\text{u}}_{0\text{j}}+e_{0ij}$$

### Adults

In these equations, the outcome for the i^th^ visitor in the j^th^ exhibit is modeled as main effect of educator condition (γ_10_) with γ_00_ as the overall mean and u_0j_ as the exhibit residuals and *e*_*0ij*_ as the individual residuals. This general equation was tested for each of the three dependent variables (Topic Interest, Perceived Learning and Total Content Questions Correct).

$$\gamma_{ij}=\gamma_{00}+\gamma_{10}EducatorCondition_{ij}+{\text{u}}_{0\text{j}}+e_{0ij}$$

## Results

### Topic interest: Youth

**Fig 1:**
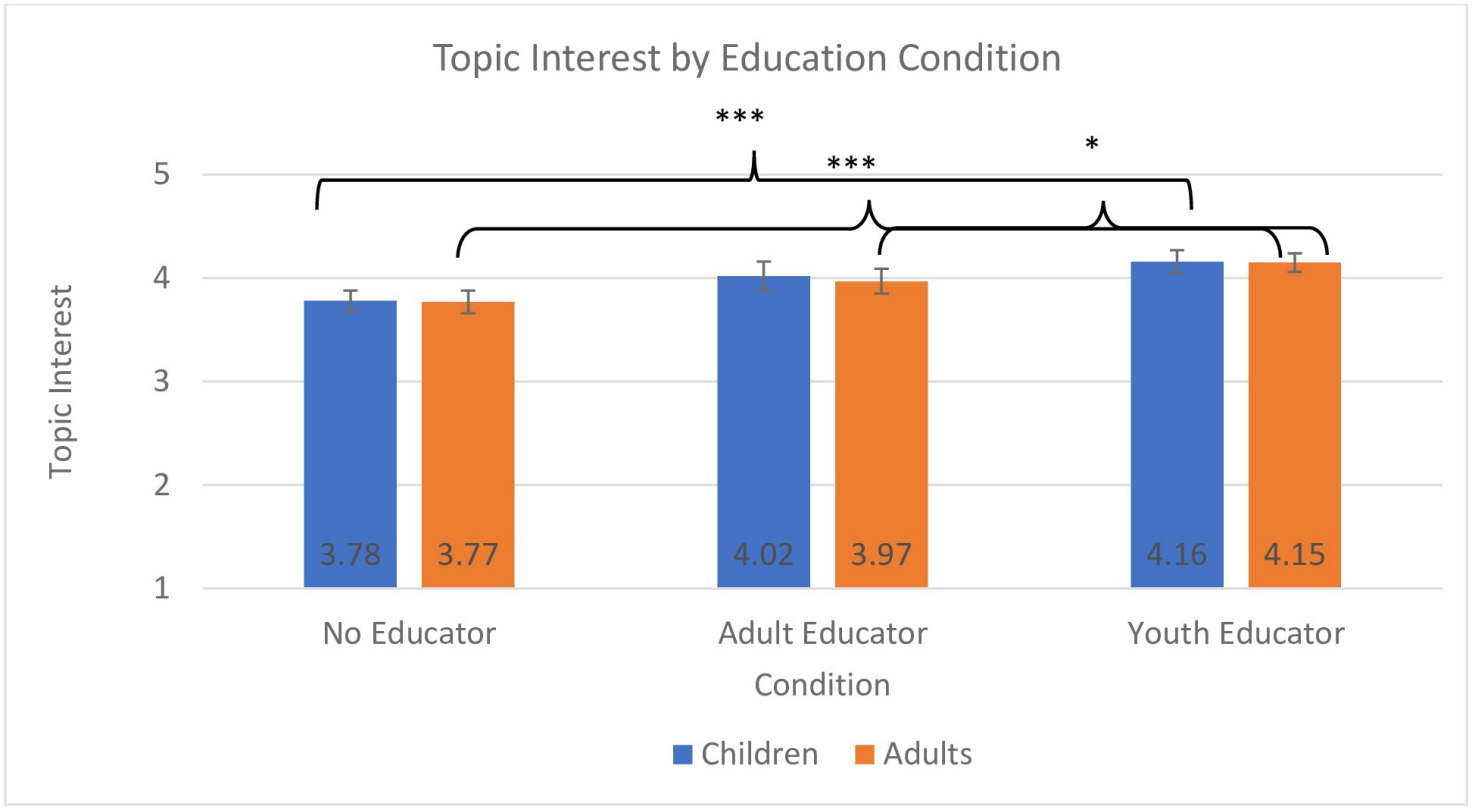
Topic interest by education condition. Analyses for children and adults were conducted separately, but are displayed in one graph for ease of interpretation across analyses; * *p* < .005, ** *p* < .01, *** *p* < .001.

**Fig 2:**
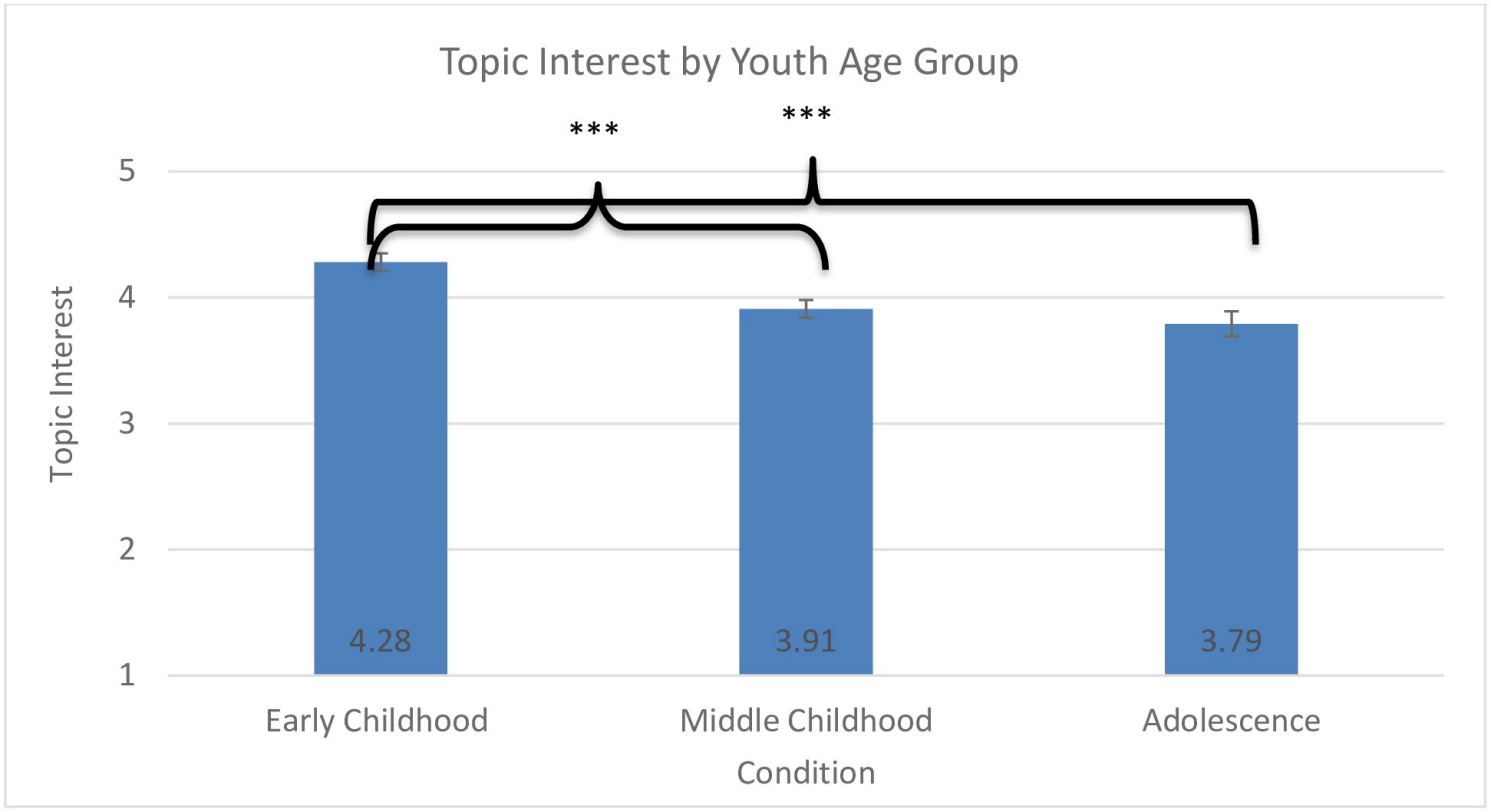
Topic interest by youth age group. * *p* < .005, ** *p* < .01, *** *p* < .001.

Confirming hypotheses 1 and 3, the model for visitor interest in the exhibit topic revealed a significant effect of educator condition on visitor interest, *F*(2, 631) = 7.54, *p* < 0.001, *η*_*p*_^*2*^ = .02, (Fig 1) and a significant effect of visitor age group on self-reported exhibit interest, *F*(2,930) = 12.40, *p* < 0.001, *η*_*p*_^*2*^ = .03 (Fig 2). In terms of the age effect, the early childhood group reported higher interest in the topic of the exhibit than did either the middle childhood (*p* < 0.001) or the adolescent group (*p* < 0.001). In terms of educator condition, youth who interacted with the static media (no educator) reported lower interest than did those who interacted with a youth educator (*p* < 0.001), but there were no differences between interacting with an adult educator and a youth educator or an adult educator or the static media.

### Topic interest: Adults

Confirming hypothesis 1, the model for visitor interest in the exhibit topic revealed a significant effect of educator condition on visitor interest, *F*(2, 1049) = 9.74, *p* < 0.001, *η*_*p*_^*2*^ = .02 (Fig 1). Adult visitors expressed greater interest when working with a youth educator than with no educator (*p* < 0.001) and greater interest when they interacted with a youth educator than an adult educator (*p* = 0.05).

### Perceived learning: Youth

**Fig 3:**
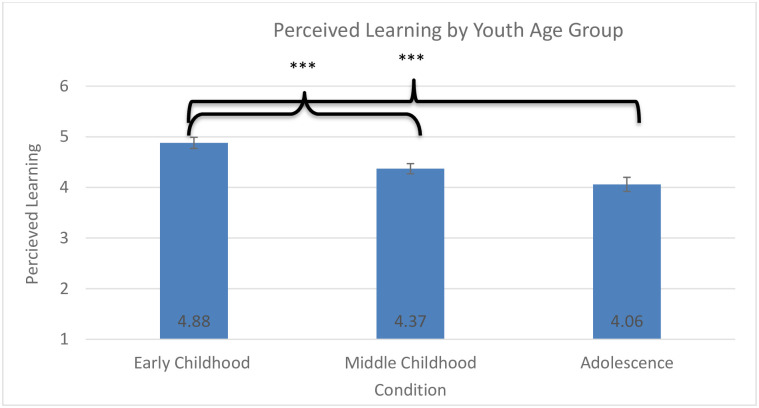
Perceived learning by youth age group. * *p* < .005, ** *p* < .01, *** *p* < .001.

**Fig 4:**
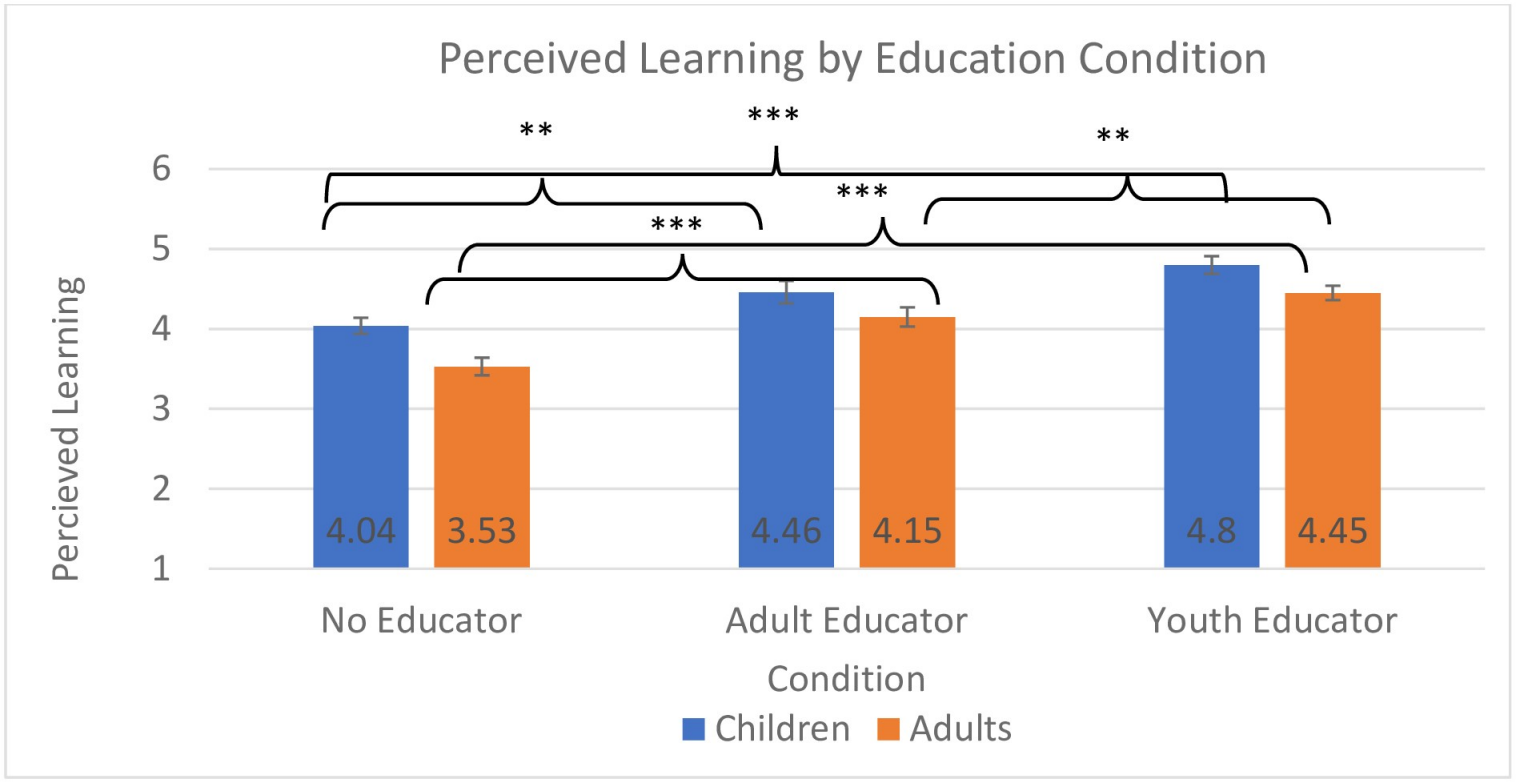
Perceived learning by education condition. Analyses for children and adults were conducted separately, but are displayed in one graph for ease of interpretation across analyses; * *p* < .005, ** *p* < .01, *** *p* < .001.

Confirming Hyp 1 and 2, the model for perceived learning revealed a significant effect of visitor age group on self-reported exhibit interest, *F*(2,933) = 17.59, *p* < 0.001, *η*_*p*_^*2*^ = .04 (Fig 3), and a significant effect of educator condition on visitor interest, *F*(2, 652) = 18.30, *p* < 0.001, *η*_*p*_^*2*^ = .05 (Fig 4). The early childhood group perceived that they learned more from the exhibit than did either the middle childhood (*p* < 0.001) or the adolescent group (*p* < 0.001). Participants who interacted with the static media (no educator) perceived that they learned less than did those who interacted with a youth educator (*p* < 0.001) or an adult educator (*p* = 0.009).

### Perceived learning: Adults

Confirming hypothesis 1, the model for perceived learning for adults revealed a significant effect of educator condition, *F*(2, 806) = 30.60, *p* < 0.001, *η*_*p*_^*2*^ = .07 (Fig 4). This revealed that adults who interacted with youth educators perceived they learned more than those who interacted with adult educators (*p* = 0.01) or no educator (*p* < 0.001). Additionally, those who interacted with an adult educator believed they learned more than those who interacted with just the exhibit (*p* < 0.001).

### Correct content questions: Youth

**Fig 5:**
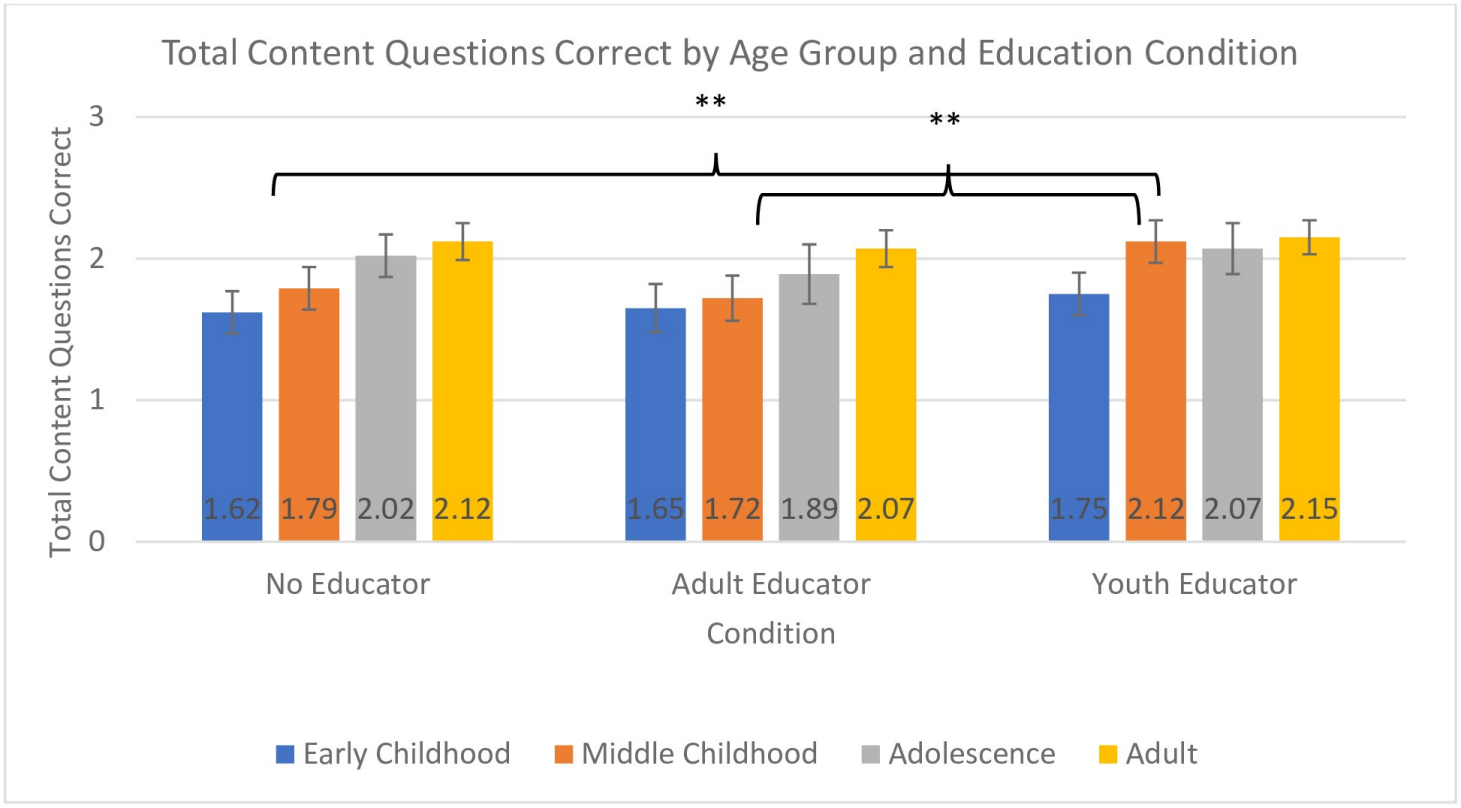
Total content questions correct by age group and condition. Analyses for children and adults were conducted separately, but are displayed in one graph for ease of interpretation across analyses; * *p* < .005, ** *p* < .01, *** *p* < .001.

Confirming hypothesis 4 and partially confirming hypothesis 5, the model for correct content questions revealed a significant effect of visitor age group on number of questions answered correctly, *F*(2,955) = 7.34, *p* < 0.001, *η*_*p*_^*2*^ = .02, but no significant effect of educator condition on number of questions answered correctly, *F*(2, 965) = 2.76, *p* = 0.064, *η*_*p*_^*2*^ = .01 (Fig 5). The early childhood group responded correctly to fewer questions than did either the middle childhood (*p* = 0.011) or the adolescent group (*p* = 0.002). While there was not a significant effect of educator condition, or the interaction between educator condition and age-group, because of the trend towards significance, we further explored the pattern in the data. This revealed that while children in early childhood and adolescence did not differ in the number of content questions they answered correctly across the educator conditions, the children in middle childhood responded to more content questions correctly if they interacted with a youth educator than if they interacted with an adult educator (*p* = 0.005) or no educator (*p* = 0.008).

### Correct content questions: Adults

Contrary to hypothesis 1, the model for adults for correct content questions revealed no differences based on educator condition, *F*(2,1140) = 0.615 (Fig 5).

## Discussion

Our results provide novel insight into the experiences of visitors in ISLS. First, we find evidence that interacting with an educator at ISLS has particular benefits. Visitors who interacted with youth or adult educators believed that they learned more than did those who interacted just with the exhibit and adult visitors saw an added benefit if they interacted with a youth educator over an adult educator in terms of perceived learning. Further, we document a particular benefit of youth educators: visitors (both youth and adults) who interacted with a youth educator rated their interest in the science topic of the exhibit higher than those who interacted with the exhibit without an educator present. In terms of developmental findings, youth in early childhood believe they learn more from the exhibits and rate their interest in the science topics covered at the exhibits as higher than youth in middle childhood and adolescence visitors. However, youth in early childhood answered fewer content questions correct than did youth in middle childhood or adolescence. Further, we found that youth in middle childhood were especially likely to benefit from interacting with a youth educator, answering more questions correctly than those who interacted with an adult educator or just the exhibit. The results provide important insights into experiences at ISLS, suggesting the importance of staffing exhibits with educators, especially youth educators, and highlighting that youth in different developmental periods may need differentiated experiences in ISLS.

An explicit goal of many ISLS is to promote interest in science content [7] and our findings suggest that topic interest was high across our participants. This is not surprising, given that our participants were visiting the ISLS during their leisure time. However, those who encountered a youth educator expressed even higher interest than those who interacted with the exhibit in the absence of an educator. Additionally, adult visitors who interacted with a youth educator expressed greater interest than those who interacted with an adult educator. Prior research has not explored adult learning from youth and adults in informal learning settings. These findings, however, suggest that youth educators are particularly effective in engaging the interest of adult visitors. This may be because adult visitors are particularly invested in engaging with youth educators because they see inherent value in learning from young people. For example, an adult visitor might be more likely to engage with and subsequently learn from a youth educator because given their younger age, adult visitors would like youth educator to feel as though their time and effort is valued and appreciated. An additional possibility is that learning from a youth educator poses less of a threat to the self-esteem of adult visitors than learning from an adult peer might. While our current measures do not allow us to explicitly explore why adult visitors reported greater interest when interacting with youth educators, these findings provide important new directions for future research. In terms of the youth visitors, previous reports on peer learning noted increased motivation and subject interest when learners interact with others close to their age [36, 37]. Thus, youth educators may be particularly likely to build interest in youth visitors, who are close to them in age. Confirming previous studies, which have also identified a decline in science interest and motivation as children age [38, 39], we also found that our youth visitors in early childhood expressed greater topic interest than other youth visitors. However, even though these differences were significant, even youth in middle childhood and adolescence expressed high topic interest (well above the scale mid-point). Thus, ISLS are engaging to visitors of all ages and future research might further explore factors such as how to encourage visitors from diverse backgrounds and developmental periods to visit ISLS, as our findings suggest that those who do visit find the science topics engaging and interesting. This is especially important as research suggest that not all families feel welcome in ISLS, with findings indicating that ethnic minority families at times perceive ISLS as “not for them” [40]. In our current study, we were unable to explore differences by participant ethnicity as our sample was primarily ethnic majority families, however this is an important area for extension in future studies.

In terms of perceived learning, participants who interacted with youth or adult educators showed significantly higher perceived learning than those who explored the exhibit without guidance from an educator. Further, adult learners also perceived that they learned more from youth educators than from adult educators. Perceived learning is related to learner self-efficacy and is associated with academic motivation and success in children [41]. As highlighted by Social Learning Theory [23], learning is enhanced when learners have opportunity for social interaction. Our findings provide support that there is a particular benefit for perceived learning from interacting with an educator in ISLS. This is an important finding as much of the prior research on learning in museums and other informal science contexts has focused largely on the impact of family talk and family interaction [17]. What these findings demonstrate is the value of staffing exhibits with educators in order to enhance learning outcomes for visitors.

While perceived learning is an important outcome, we also find differences in correct responses to content questions about the exhibits in our youth sample. Developmentally, our early childhood participants perceived that they learned significantly more than did participants in middle childhood or adolescence, but they actually responded to fewer questions correctly than did either of our older age groups. This is not all that surprising, as young children often over estimate their competency [30], but it does indicate the value of ensuring that education provided at ISLS is developmentally differentiated. This is a challenge as visitors to these sites come from all ages and at varying levels of prior knowledge. However, prior research does suggest that social partners are able to assess the expertise and prior knowledge of others as they share information with them [42] the role that the educators may have taken. Future research might explicitly measure the ability of educators to gauge the prior knowledge of the learners with whom they interact and identify possible trainings to foster greater attunement to the learner’s level of prior knowledge.

While our data suggest a trend towards learning more from youth educators than from adult educators or just the exhibit alone, there was only a statistically significant benefit to interacting with a youth educator over the other conditions for youth in middle childhood. This may be because youth educators (who were all adolescents) are able to serve most effectively as more knowledgeable others for peers in middle childhood, as they will be able to make prior relevant connections but also to push learners in middle childhood further in their learning through scaffolding. Thus, children in middle childhood may be in an optimal zone for youth educators to be particularly beneficial. It may be that adolescents do not perceive youth educators, who are likely very close to the adolescent visitors in terms of age, as experts or more knowledgeable than themselves. Young children may be less able to connect with youth educators who could be too far removed in terms of shared experiences. Future research should continue to explore why the same benefits for youth educators were not found for all participants.

### Limitations and future directions

Our research provides important insight into the learning context in ISLS. However, further insight using observations of family units visiting these sites, would help to clarify what additional factors can ensure an optimal learning environment. Further, our data do not clarify what the role of the parents or other visitors as educators or co-educators in these interactions is. For example, parents may step in and provide relevant prior connections to the exhibit content in the absence of an educator. Prior research has documented the rich nature of parent-child talk in informal science learning sites [17]. However, research has also shown that content-related talk is often longer when it occurs between visitors and staff than when it is between visitors only [43]. Observing family units as they navigate the exhibits may provide deeper insight into how the experience of visiting an exhibit is different with an educator present than when one is not present. Visitors are more likely to approach an exhibit (a primate zoological exhibit, in this case) and reported greater perceived learning when a scientist was present [44]. However, if they interacted with exhibit signage, visitors reported greater knowledge and understanding of the information on those signs [44]. These findings suggest that static media may provide accurate information, but that engagement may be higher when an educator or scientist is present at the exhibit.

In the current study, we used simple measures of learning and interest. As an example, the content questions employed were designed at the knowledge/recall level of Bloom’s taxonomy [45]. This level of understanding was appropriate for examining the outcomes of brief interactions in ISLS, however, further study should include higher-level questions to examine deeper aspects of understanding about the topics being presented. It would also be important for future research to aim to examine retention of content over time, for instance by following visitors longitudinally. This would be challenging with ISLS visitors but would provide insight into learning over a longer time period.

## Conclusions

Our findings document the benefits of visits to ISLS, highlighting the important role that educators play in these settings. Globally, museums spend over $2 billion dollars a year on education and provide more than 18 million hours of instruction yearly as part of their programming [46]. Our findings support the use of ISLS educational funding for youth and adult educators. Thus, the results also have implications for policy and practice in ISLS, suggesting that funding educator positions, especially for youth educators, is likely to provide measurable benefits for visitors. This research further emphasizes the heterogeneity of visitor experiences, revealing that children, adolescents and adults who visit these sites have distinct experiences with young children showing the greatest interest and perceptions of their learning but also exhibiting less recall of the content taught at these exhibits. This suggests that ISLS may benefit from differentiating the education provided (both provided by the educators and as part of the exhibit itself) depending on the age of the visitors.

## Supporting information

S1 File Adult learning paper data. (SAV)

S2 File Children learning paper data. (SAV)

S3 File Combined consent procedures 20Feb20. (DOCX)

S4 File Notification letter visitor data collection STEM teens. (DOCX)
