# Supplementary material for: Interest and learning in informal science learning sites: Differences in experiences with different types of educators
Source: PLoS One. 2020 Jul 23;15(7):e0236279. doi: 10.1371/journal.pone.0236279 (PMC7377401; doi:10.1371/journal.pone.0236279)
Supplement: S3 File — (DOCX) [file pone.0236279.s003.docx]

Child Assent Procedure:

What's it about?

If you are happy to help us today, we will ask you to answer some questions about what you think about science museums and learning about science. We are interested in how **you**feel about your visit to this science centre today.

Will anyone know what I say?
All your answers are completely **confidential and anonymous**. This means we do not ask for your name, and we do not share your answers with anyone. We are really interested in what **you** think!

How long will it take?
The questionnaire is **quick and easy** to do, just put down your first thought for each question. You can **stop at any time**, and do not have to give a reason. If there are any questions you **don't** want to answer, you can leave them out.

Remember, this is**not a test**, there are no wrong answers, so please write whatever you think.

Please tick below to show if you want to help or don't want to help.


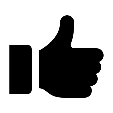


I am happy to take part


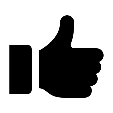


I don’t want to take part

Parent Consent Procedure

Hello and welcome to this survey. Here, we are trying to understand how people who visit

science centres, zoos, and aquariums learn about and engage with science.

This survey will ask you some questions about your visit to this science centre. This is not a

test and there are no right or wrong answers.

In line with GDPR laws in the EU we take the protection of your data very seriously. All data

will be held in a password-secured and encrypted file that is only accessible to trained

researchers.

Your data will never be analysed individually, we will only look at all responses together. We

may go on to present this data at scientific conferences or in academic papers.

Your data will be securely and confidentially stored for 5 years after collection.

If there are any questions you don't feel comfortable answering you can leave them blank. If

you wish to stop at any time please simply hand in the survey.

I understand that my participation is voluntary and that I am free to withdraw at any time

without giving any reason and without my legal rights being affected

***Yes, I understand***

I understand that relevant sections of the data collected may be looked at by members of

the research team from Goldsmiths and North Carolina State University, where it

is relevant. I give permission for these individuals to have access to my records

***Yes, I give permission***

I understand that taking part involves an anonymous questionnaire to be used for reports

published in academic journals

***Yes, I understand***

I understand that my child is taking part in a survey that is also anonymous and confidential.

All data from my child’s survey will be stored securely and anonymously in an encrypted file

for 5-years.

***Yes I understand and give permission for my child to take part***

Please select one of the following options:

**I have read the above information and I am happy to take part in the survey**

**I have read the above information and I do not want to take part in the survey**
