# Supplementary material for: Interest and learning in informal science learning sites: Differences in experiences with different types of educators
Source: PLoS One. 2020 Jul 23;15(7):e0236279. doi: 10.1371/journal.pone.0236279 (PMC7377401; doi:10.1371/journal.pone.0236279)
Supplement: S4 File — (DOCX) [file pone.0236279.s004.docx]

**Assessing the Impact of Informal Science Learning Sites**

Dear Visitor,

You and your child(ren), if applicable, are invited to participate in a study examining how much visitors learn from informal science learning sites. We are professors at the University of South Carolina, North Carolina State University, and Goldsmiths University of London. We are working on a project related to understanding the impact of informal science learning sites with a focus on youth programming.

We are asking to include you (and your child(ren), if applicable) in this study because you are visiting an informal science learning site, such as a museum, zoo or aquarium. We expect to have 1000 participants in the study. If you agree to participate, you (and your child(ren), if applicable) will complete a survey (10 minutes) about your experiences and interest with science, mathematics, and your visit today. We will also send you a follow-up survey after 3 months and 6 months. Families will be compensated with a $5 gift card for each survey they complete.

Participation will be confidential. A number will be assigned to each participant at the beginning of the project. Study records/data will be stored in locked filing cabinets and protected computer files. Responses to questions will not be linked to you or your child’s name. The results of the study may be published or presented at professional meetings, but your identity or your child’s identity will not be revealed.

If you have any questions, please email me us at [stemteens@sc.edu](about:blank) .

Sincerely,

Dr. Adam Hartstone-Rose

Dr. Kelly Lynn Mulvey

Dr. Matthew J. Irvin

Dr. Eric Goff
